# The Multifaceted Effects of Polysaccharides Isolated from *Dendrobium huoshanense* on Immune Functions with the Induction of Interleukin-1 Receptor Antagonist (IL-1ra) in Monocytes

**DOI:** 10.1371/journal.pone.0094040

**Published:** 2014-04-04

**Authors:** Juway Lin, Ya-Jen Chang, Wen-Bin Yang, Alice L. Yu, Chi-Huey Wong

**Affiliations:** 1 Institute of Biochemical Sciences, National Taiwan University, Taipei, Taiwan; 2 Genomics Research Center, Academia Sinica, Taipei, Taiwan; 3 Institute of Biomedical Sciences, Academia Sinica, Taipei, Taiwan; 4 Department of Pediatrics/Hematology-Oncology, University of California San Diego Medical Center, San Diego, California, United States of America; 5 Center of Stem Cell & Translational Cancer Research, Chang Gung Memorial Hospital, Taoyuan, Taiwan; 6 The Scripps Research Institute, La Jolla, California, United States of America; University of San Francisco, United States of America

## Abstract

*Dendrobium huoshanense* is a valuable and versatile Chinese herbal medicine with the anecdotal claims of cancer prevention and anti-inflammation. However, its immunological activities are limited to *in vitro* studies on a few cytokines and immune cell functions. First, we investigated the effects of polysaccharides isolated from DH (DH-PS) on inducing a panel of cytokines/chemokines in mice *in vivo* and human *in vitro*. We found that DH polysaccharides (DH-PS) induced TH1, TH2, inflammatory cytokines and chemokines in mouse *in vivo* and human cells *in vitro.* Secondly, we demonstrated that DH-PS expanded mouse splenocytes *in vivo* including CD4^+^ T cells, CD8^+^ T cells, B cells, NK cells, NKT cells, monocytes/macrophages, granulocytes and regulatory T cells. Notably, DH-PS induced an anti-inflammatory molecule, IL-1ra, in mouse and human immune cells, especially monocytes. The serum level of IL-1ra elicited by the injection of DH-PS was over 10 folds of IL-1β, suggesting that DH-PS-induced anti-inflammatory activities might over-ride the inflammatory ones mediated by IL-1β. The signaling pathways of DH-PS-induced IL-1ra production was shown to involve ERK/ELK, p38 MAPK, PI3K and NFκB. Finally, we observed that IL-1ra level induced by DH-PS was significantly higher than that by F3, a polysaccharide extract isolated from another popular Chinese herbal medicine, *Ganoderma lucidum*. These results indicated that DH-PS might have potential applications for ameliorating IL-1-induced pathogenic conditions.

## Introduction

*Dendrobium huoshanense* (DH), which is an herb of Orchidaceae family, has been used as a traditional Chinese herbal medicine for centuries with the anecdotal claims of cancer prevention and anti-inflammation. Polysaccharides isolated from *Dendrobium huoshanense* have been reported to induce TNF-α in peritoneal macrophages and IFN-γ in mouse splenocytes [1] and promote phagocytosis of macrophages [2]. To date, there have been no detailed studies on the systemic immune functions of DH-PS such as *in vivo* immune cell activations, inductions of comprehensive panel of cytokines/chemokines and anti-inflammatory molecules.

Among the cytokines, two forms of Interleukin-1 (IL-1α and IL-1β) are thought to play an important role in inflammation and involved in many pathological conditions including rheumatoid arthritis [3], [4]. They are produced primarily by mononuclear phagocytes, but also by a number of other cell types including skin keratinocytes [5]. These two cytokines are pro-inflammatory cytokines which can stimulate the expressions of genes associated with inflammation and autoimmune diseases. IL-1 exerts its functions by binding to type Ι IL-1 receptor and induces downstream signaling, leading to the expressions of numerous genes resulting in inflammation [6], [7], [8], [9]. A natural inhibitor of IL-1 activity, designated as secreted Interleukin-1 receptor antagonist (IL-1ra), was discovered and purified from the urine of the patients suffering from monocytic leukemia [7], [10]. IL-1ra, a 25 KD glycoprotein, is a member of IL-1 family that competes with IL-1 for the binding to IL-1 receptor, but unlike IL-1, this binding does not induce any signal transduction [11], [12], [13], [14]. IL-1ra is released *in vivo* during inflammation and immune-mediated diseases [15], which is thought to limit the deleterious effects brought by IL-1 [16], [17] and shown to be effective in the treatment of sepsis, graft-versus-host disease and rheumatoid arthritis in animal models [18], [19], [20], [21]. Additionally, IL-1ra (commercially produced as “anakinra”) has been used clinically to treat rheumatoid arthritis in which IL-1 plays a key role [22]. Many types of immune cells are reported to secrete IL-1ra including neutrophils, master cells, macrophages and monocytes [23], [24], [25] and several molecules have been shown to stimulate the secretion of IL-1ra including cytokines (IL-6 and IL-10, for example) and natural products [13], [26]. Polysaccharides isolated from *Ganoderma Lucidum*, another popular Chinese herbal medicine, have been shown to possess immune-modulating effects and reported to induce the production of IL-1ra [27]. Although DH-PS is believed to possess immune-modulating functions with the anecdotal claims of cancer prevention and anti-inflammation, comprehensive information regarding DH-PS on immune functions and its ability to stimulate the secretion of IL-1ra is still lacking.

In the present study, we evaluated the immune-modulating effects of DH-PS by investigating the profiles and kinetics of cytokine productions, as well as expansions and activations of immune cells induced by DH-PS in both human and mouse system. Moreover, we demonstrated that DH-PS induced IL-1ra production through the activation of PI3K, p38 MAPK and NFκB. Finally, we compared the IL-1ra production induced by DH-PS and F3, the polysaccharide extract from another popular Chinese herbal medicine, *Ganoderma lucidum*.

## Results

### DH-PS induced the secretions of multiple cytokines and chemokines *in vivo*

**Figure 1:**
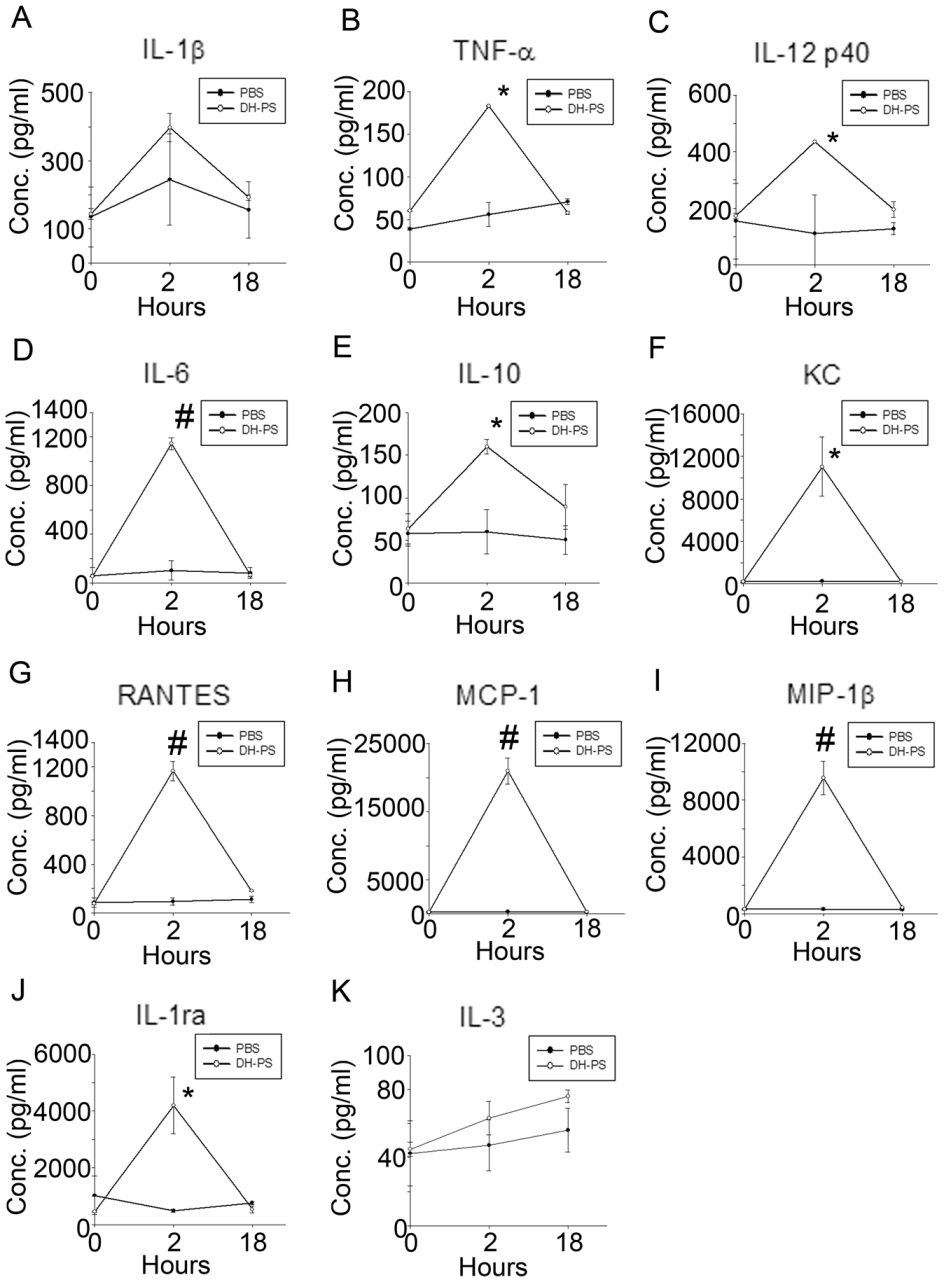
DH-PS elicited the productions of cytokines and chemokines *in vivo*. BALB/c mice (n = 3 for DH-PS and PBS group) were injected intraperitoneally with DH-PS (300 μg/mouse) or PBS only. Sera collected at 0 (before injection), 2 and 18 hours were used for the measurements of cytokines and chemokines. Y-axis represented the mean concentrations (Conc.) of cytokines/chemokines with error bars showing the standard deviation of three mice. Statistically significant difference: * compared with PBS-treated group, p<0.01. # compared with PBS-treated group, p<0.001.

In order to understand the immunological activities of DH-PS, we investigated the profiles of cytokines and chemokines induced by DH-PS in mice. BALB/c mice (n = 3 for DH-PS and phosphate buffered saline (PBS) group) were injected intraperitoneally (IP) with DH-PS (300 μg/mouse) in PBS or PBS only and the sera were collected at 0 (before injection), 2 and 18 hrs after the injection of DH-PS for the detection of cytokines and chemokines, which were quantified by Beadlyte Mouse 21-plex Cytokine Detection system and ELISA kits (for IL-1ra). Compared with PBS group shown in Figure 1, the increase of TNF-α, IL-12 p40, IL-6, IL-10 and RANTES were observed at 2 hrs, along with robust inductions of KC, MCP-1 and MIP-1β. An apparent increase in IL-1β at 2 hrs was noted but it did not reach statistical significance. These results suggested that DH-PS induced Th1 (IL-12 p40), Th2 (IL-6, IL-10), inflammatory cytokines (TNF-α) and chemokines (KC, RANTES, MCP-1, MIP-1β), which might further modulate the downstream activations of immune cells. Furthermore, an anti-inflammatory molecule, IL-1ra, was increased from 465 to 4199 pg/ml (∼9 folds of PBS control group) at 2 hrs. This indicated that the amounts of IL-1ra induced were 10 folds more than IL-1β (increased from 144 to 397 pg/ml, ∼1.6 folds of PBS control group), suggesting that DH-PS-induced IL-1ra might over-ride the activity induced by IL-1β.

### DH-PS expanded and activated subpopulations of immune cells *in vivo*

**Figure 2:**
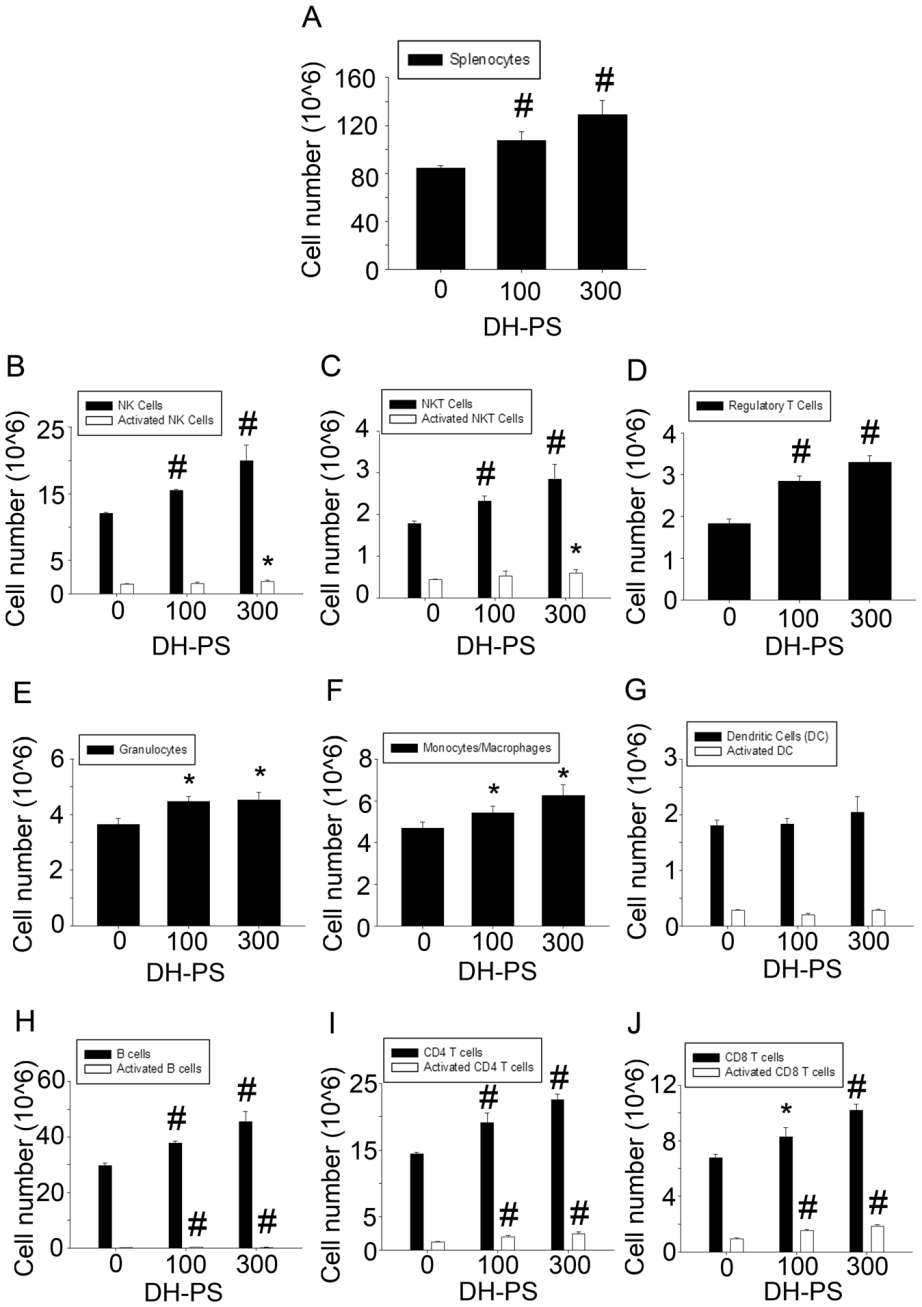
DH-PS expanded subpopulations of splenocytes. BALB/c mice (n = 3 for DH-PS and PBS group) were injected intraperitoneally with DH-PS (100 μg or 300 μg) or PBS only (DH-PS  = 0) and sacrificed at 72 hrs for the harvest of splenocytes. Cell populations were characterized by the following markers: NK cells (NK-1.1^+^CD3^−^), activated NK cells (NK-1.1^+^CD3^−^CD69^+^), NKT cells (NK-1.1^+^CD3^+^), activated NKT cells (NK-1.1^+^CD3^+^CD69^+^), regulatory T cells (CD4^+^CD25^+^FOXP3^+^), granulocytes (Ly6G^+^), monocytes and macrophages (CD11b^+^), dendritic cells (CD11c^+^), activated dendritic cells (CD11C^+^CD80^+^CD86^+^), B cells (B220^+^CD23^+^), activated B cells (B220^+^CD23^+^CD69^+^), CD4^+^ T cells (CD3^+^CD4+), activated CD4^+^ T cells (CD3^+^CD4^+^CD69^+^), CD8^+^ T cells (CD3^+^CD8^+^) and activated CD8^+^ T cells (CD3^+^CD8^+^CD69^+^). Results were presented as mean values with error bars showing the standard deviation of three mice. Results were converted to log value before the determination of statistical significance. Statistically significant difference: * compared with PBS-treated group, p<0.05. # compared with PBS-treated group, p<0.01.

In order to investigate the effects of DH-PS on immune cells, we further analyzed whether DH-PS expanded and/or activated the subpopulations of mouse splenocytes. BALB/c mice (n = 3 for DH-PS and PBS group) were injected intraperitoneally with DH-PS (100 μg or 300 μg) or PBS (DH-PS = 0) only and sacrificed at 72 hrs for the harvest of splenocytes. Cells were counted and analyzed for markers characteristic of specific subpopulations of splenocytes by flow cytometry to determine the expansions/activations of various immune effector cells. As shown in Figure 2A, in comparison with PBS control group, the number of splenocytes was increased after the administration of DH-PS in a dose-dependent manner. Subpopulations of splenocytes examined included innate immune cells such as natural killer cells (NK-1.1^+^/CD3^−^)/activated natural killer cells (NK-1.1^+^/CD3^−^/CD69^+^), natural killer T cells (NK-1.1^+^/CD3^+^)/activated natural killer T cells (NK-1.1^+^/CD3^+^/CD69^+^), regulatory T cells (CD4^+^/CD25^+^/Foxp3^+^), granulocytes (Ly6G^+^), monocytes and macrophages (CD11b^+^) and dendritic cells (CD11c^+^/CD80^+^/CD86^+^) and adaptive immune cells such as B cells (B220^+^CD23^+^)/activated B cells (B220^+^CD23^+^CD69^+^), CD4^+^ T cells (CD3^+^CD4+)/activated CD4^+^ T cells (CD3^+^CD4^+^CD69^+^), CD8^+^ T cells (CD3^+^CD8^+^)/activated CD8^+^ T cells (CD3^+^CD8^+^CD69^+^). Overall, DH-PS induced modest increases (up to 2.08 fold) in all subpopulations examined. Compared with PBS group, we found that DH-PS induced 1.3 and 1.7 fold increases of NK cells at 100 μg and 300 μg, respectively. A mild increase (1.24 folds) of activated NK cells was also observed at 300 μg (Fig.2B). DH-PS induced 1.3 and 1.6 fold increases of NKT cells at 100 μg and 300 μg, respectively. An increase (1.37 folds) of activated NKT cells was also observed at 300 μg (Fig.2C). DH-PS also induced 1.55 and 1.79 fold rise of regulatory T cells at 100 μg and 300 μg, respectively (Fig.2D) and a trend in increases of granulocytes and monocytes/macrophages (Fig.2E, F). However, there was no significant effect of DH-PS on dendritic cells (Fig.2G). For the adaptive immune cells, DH-PS induced 1.27 and 1.53 fold increases of B cells and 2.08 and 1.91 fold rise of activated B cells at 100 μg and 300 μg, respectively (Fig.2H). As to CD4^+^ T cells (Fig.2I), DH-PS induced 1.32 and 1.56 fold increases at 100 μg and 300 μg, respectively. Activated CD4^+^ T cells were also augmented to 1.64 and 1.98 folds, respectively. For CD8^+^ T cells (Fig.2J), DH-PS induced 1.22 and 1.50 fold increases and activated CD8^+^ T cells to 1.63 and 1.96 folds at 100 μg and 300 μg, respectively. These findings indicated that DH-PS modestly expanded and/or activated many types of immune cells in mice.

### DH-PS induced the productions of multiple cytokines and chemokines in human immune cells

**Figure 3:**
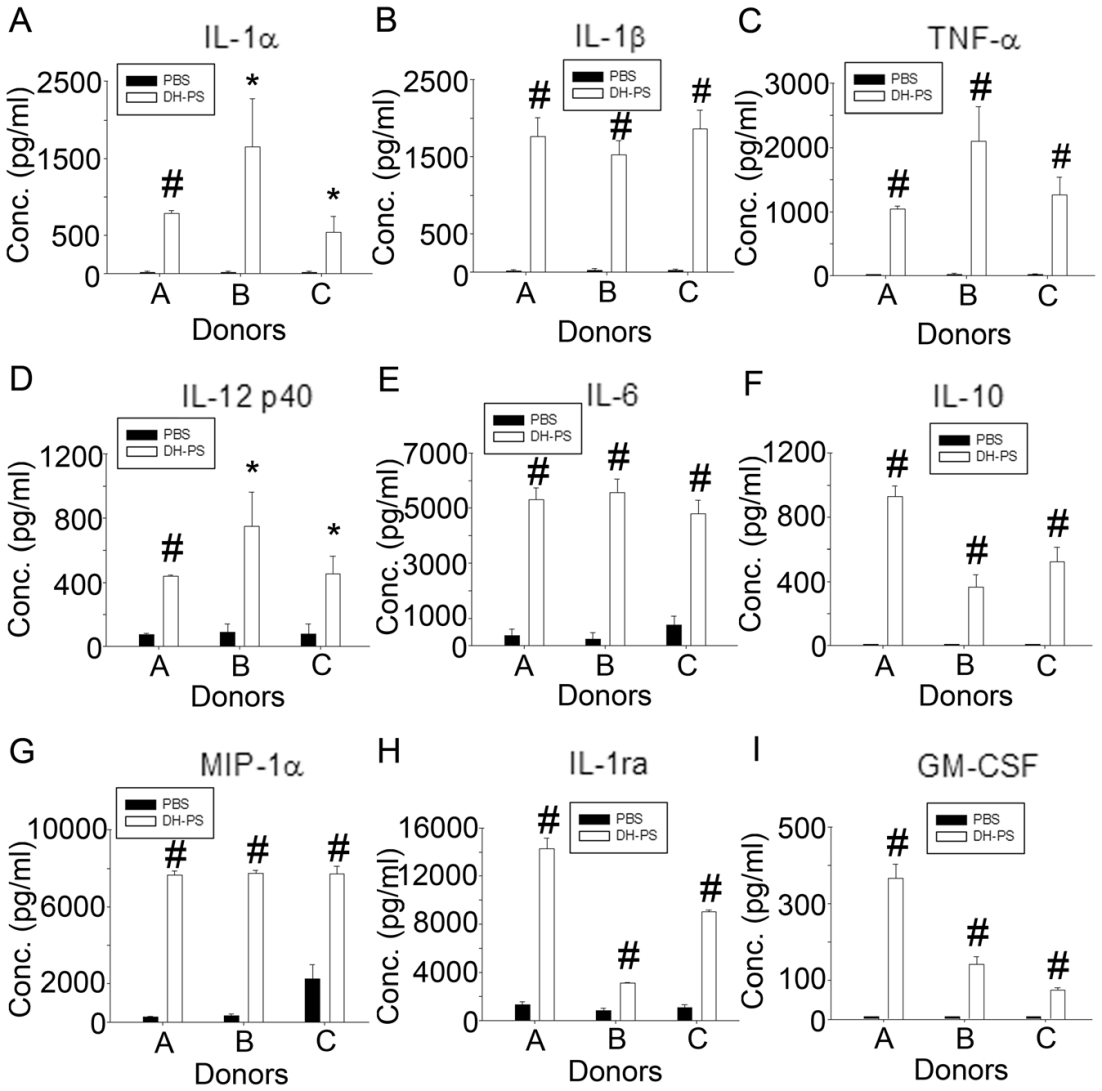
DH-PS elicited the productions of cytokines and chemokines in human peripheral blood mononuclear cells (PBMC). Human PBMCs isolated from three healthy donors were cultured (2×10^6^ cells/ml) with DH-PS (50 μg/ml) or PBS as control for 18 hrs. Supernatants were harvested for the measurements of cytokines and chemokines. Y-axis represented the mean concentrations (Conc.) of cytokines/chemokines with error bars showing the standard deviation of triplicate. Statistically significant difference: * compared with PBS-treated group, p<0.05. # compared with PBS-treated group, p<0.005.

**Figure 4:**
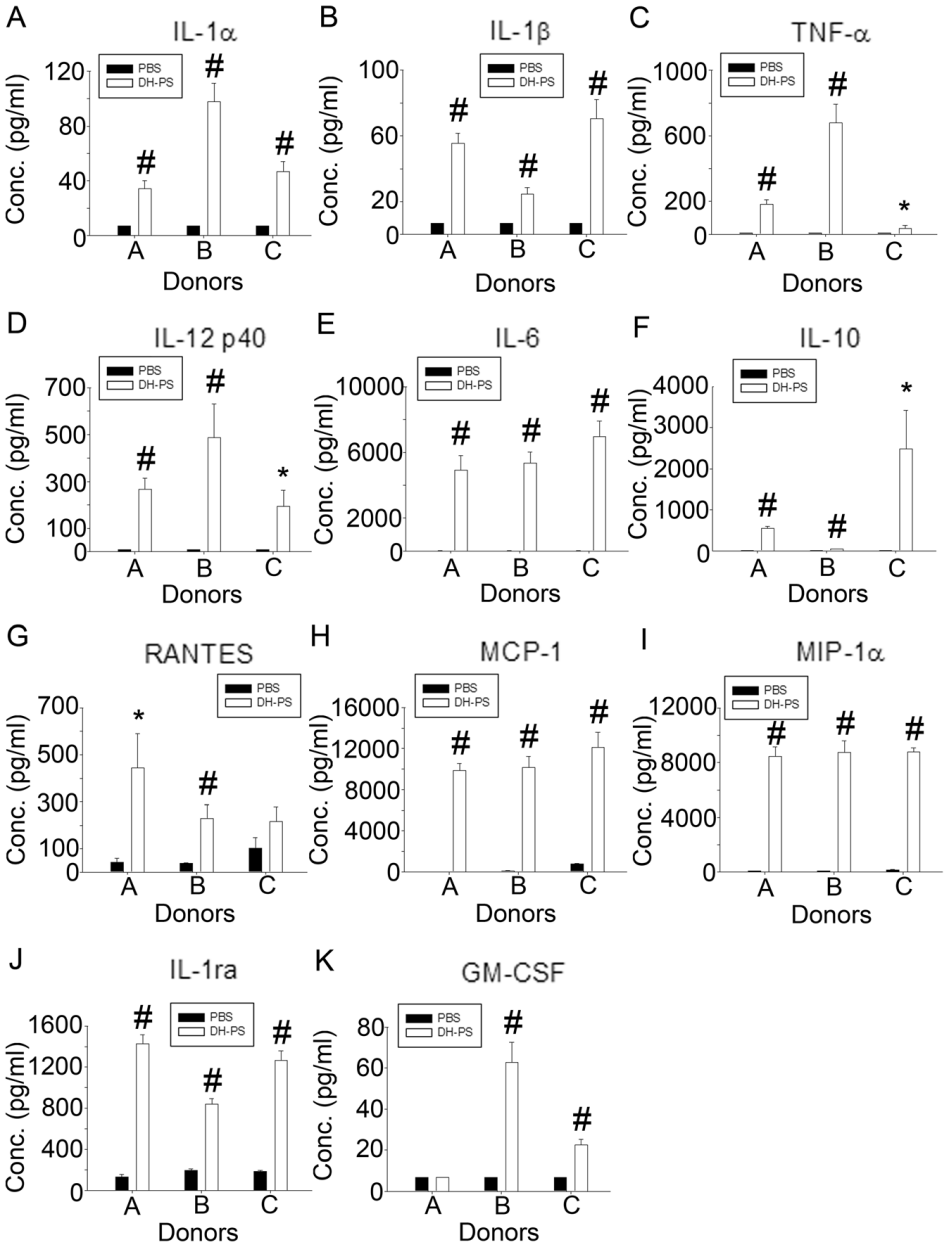
DH-PS elicited the productions of cytokines and chemokines in human CD14^+^cells. Human CD14^+^cells isolated from three healthy donors were cultured (1×10^6^ cells/ml) with DH-PS (50 μg/ml) or PBS as control for 18 hrs. Supernatants were harvested for the measurements of cytokines and chemokines. Y-axis represented the mean concentrations (Conc.) of cytokines/chemokines with error bars showing the standard deviation of triplicate. Statistically significant difference: * compared with PBS-treated group, p<0.05. # compared with PBS-treated group, p<0.005.

We next investigated the effects of DH-PS on primary human immune cells. Human peripheral blood mononuclear cells (PBMC) were isolated from three healthy donors and cultured with DH-PS (50 μg/ml) or PBS as control for 18 hrs. Supernatants were collected for the measurements of cytokines, chemokines and IL-1ra. As shown in Figure 3, TH1 (IL-12 p40), TH2 (IL-6, IL-10), inflammatory cytokines (IL-1α, IL-1β TNF-α) and chemokines (MIP-1α) were induced in PBMC by DH-PS. A growth factor GM-CSF was also augmented by DH-PS. Consistent with mouse data, IL-1ra was induced by DH-PS in human PBMC. Since PBMC contained a mixture of immune cells, we next focused on the DH-PS-induced effects on CD14^+^ cells which were reported to play a key role in rheumatoid arthritis. CD14^+^ cells were isolated from PBMC and cultured with DH-PS (50 μg/ml) or PBS as control for 18 hrs. The supernatants were collected for the measurements of cytokines, chemokines and IL-1ra. As shown in Figure 4, there were significant increases in IL-1α, IL-1β and IL-1ra along with IL-12 p40, IL-6, TNF-α, IL-10, GM-CSF, RANTES, MCP-1 and MIP-1α. Moreover, DH-PS boosted IL-1ra production from basal levels of (135–196) to 1427, 837 and 1264 (pg/ml) and induced IL-1β from 7 to 55, 24 and 70 (pg/ml) in CD14^+^ cells of 3 healthy donors. Thus the calculated IL-1ra/IL-1β ratios were 25.9, 34.8 and 18.1, suggesting that the IL-1ra induced by DH-PS might over-ride IL-1β activities. These data indicated that DH-PS induced Th1, Th2, inflammatory cytokines, chemokines and an anti-inflammatory molecule, IL-1ra, with an overall anti-IL-1β activity and the pattern of cytokines and chemokines induced by DH-PS was similar to the results in mice.

### Dose-dependency of IL-1ra induction by DH-PS in mice

**Figure 5:**
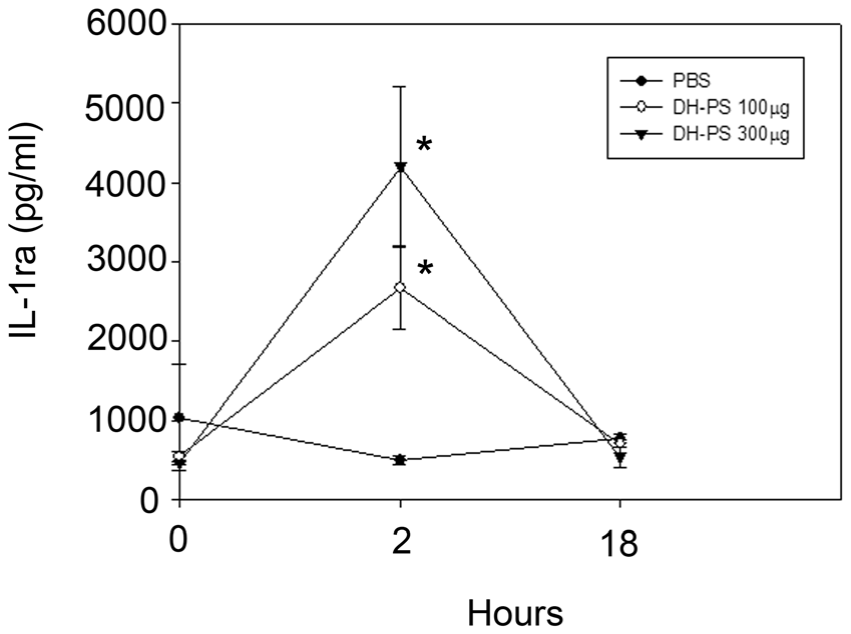
DH-PS induced IL-1ra production *in vivo*. BALB/c mice (n = 3 for each group) were injected intraperitoneally with DH-PS (100 or 300 μg/mouse) or PBS only. Sera collected at 0 (before injection), 2 and 18 hours were used for the measurements of IL-1ra by ELISA assay. Results were presented as mean concentrations with error bars showing the standard deviation of three mice. Statistically significant difference: * compared with PBS-treated group, p<0.05.

As a potent antagonist of the biological functions of IL-1, IL-1ra has been in clinical use for the treatment of IL-1-induced pathogenic conditions including rheumatoid arthritis. To further delineate the kinetics and dose effects of DH-PS on IL-1ra production, BALB/c mice (n = 3 for each group) were injected intraperitoneally with DH-PS (100 or 300 μg/mouse) or PBS. Sera were collected at 0 (before injection), 2 and 18 hours for the measurements of IL-1ra. As shown in Figure 5, IL-1ra was significantly increased in a dose-dependent manner at 2 hrs after the administration of DH-PS, as compared to PBS group, and declined rapidly to basal level at 18 hrs.

### DH-PS induced IL-1ra secretion in human monocytes but not neutrophils

**Figure 6:**
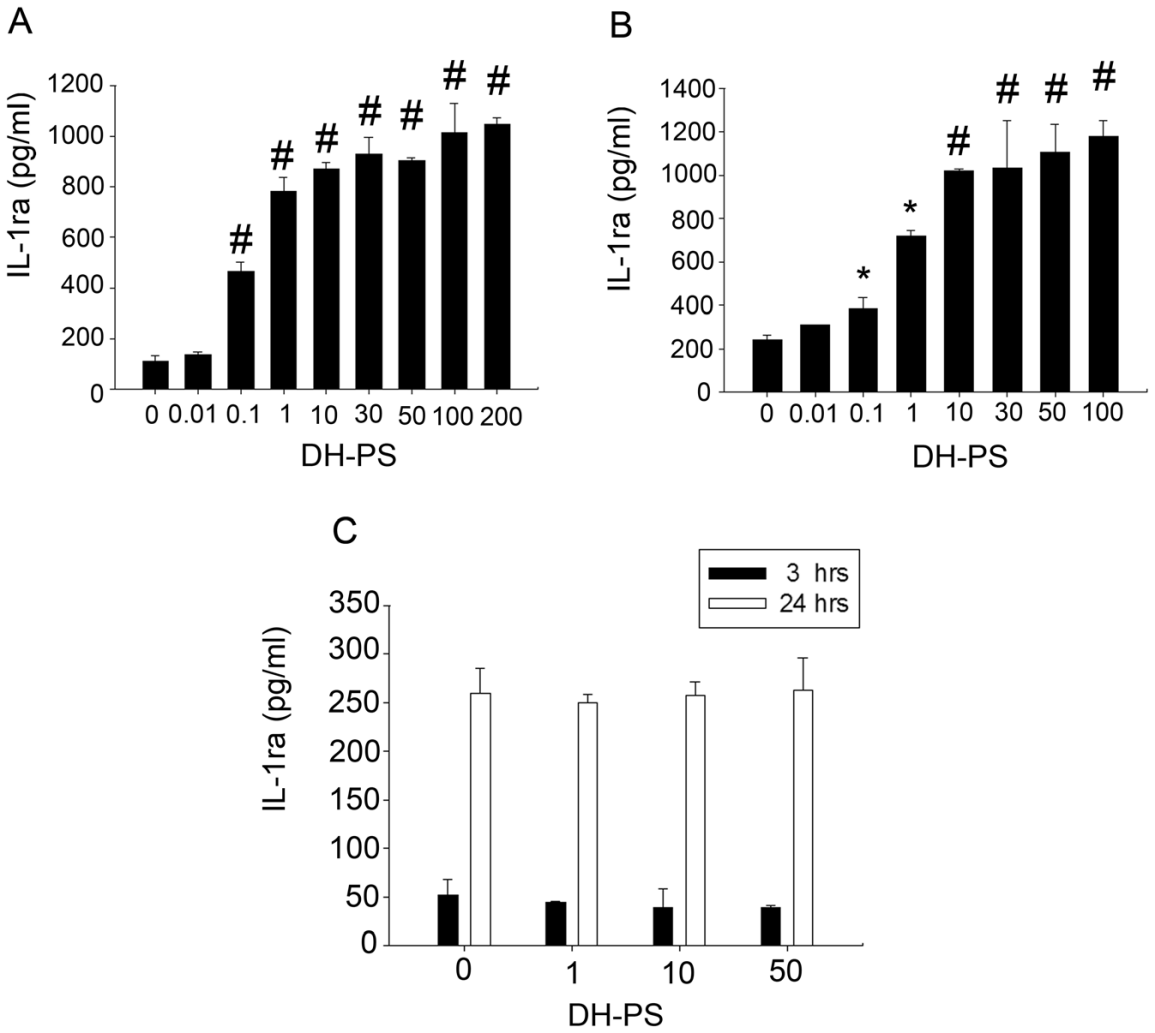
DH-PS induced IL-1ra production in human PBMC, monocytes, but not neutrophils. (A) Human PBMCs were cultured (2×10^6^ cells/ml) with increasing concentrations of DH-PS for 18 hrs and the supernatants were harvested for IL-1ra measurements. (B) Human monocytes (CD14^+^ cells) were cultured (1×10^6^ cells/ml) with increasing concentrations of DH-PS for 18 hrs and the supernatants were harvested for IL-1ra measurements. (C) Neutrophils were cultured (1×10^5^ cells/ml) with the increasing concentrations of DH-PS for 3 or 24 hours and supernatants were harvested for IL-1ra measurements. Y-axis represented the mean concentration of IL-1ra (pg/ml). X-axis represented the concentration of DH-PS (μg/ml). Concentration 0 represented the use of PBS only as vehicle control. Results were presented as mean values with error bars showing the standard deviation of triplicate. Statistically significant difference: * compared with PBS-treated group, p<0.05. # compared with PBS-treated group, p<0.005.

Next, we investigated the effects of DH-PS on IL-1ra secretion in human primary immune cells. At the concentrations ranging from 0 (use of PBS as control) to 200 μg/ml, DH-PS induced dose-dependent increases of IL-1ra production in PBMC, reaching the maximal level of ∼10 fold rise at 100 μg/ml (Fig.6A). Since monocytes and neutrophils were two types of immune cells reported to produce IL-1ra upon stimulations [24], we further investigated the responses of the two types of cells stimulated by DH-PS. CD14^+^ cells were isolated from PBMC and cultured with increasing concentrations of DH-PS ranging from 0 (use of PBS as control) to 100 μg/ml for 18 hrs. As shown in Figure 6B, DH-PS induced CD14^+^ cells to produce IL-1ra dose-dependently, reaching the maximal level of ∼6 fold increases at 100 μg/ml. However, neutrophils showed no significant production of IL-1ra with the stimulation of DH-PS for 3 or 24 hrs (Fig.6C). Taken together, DH-PS stimulated IL-1ra production in human CD14^+^ cells but not neutrophils.

**Figure 7:**
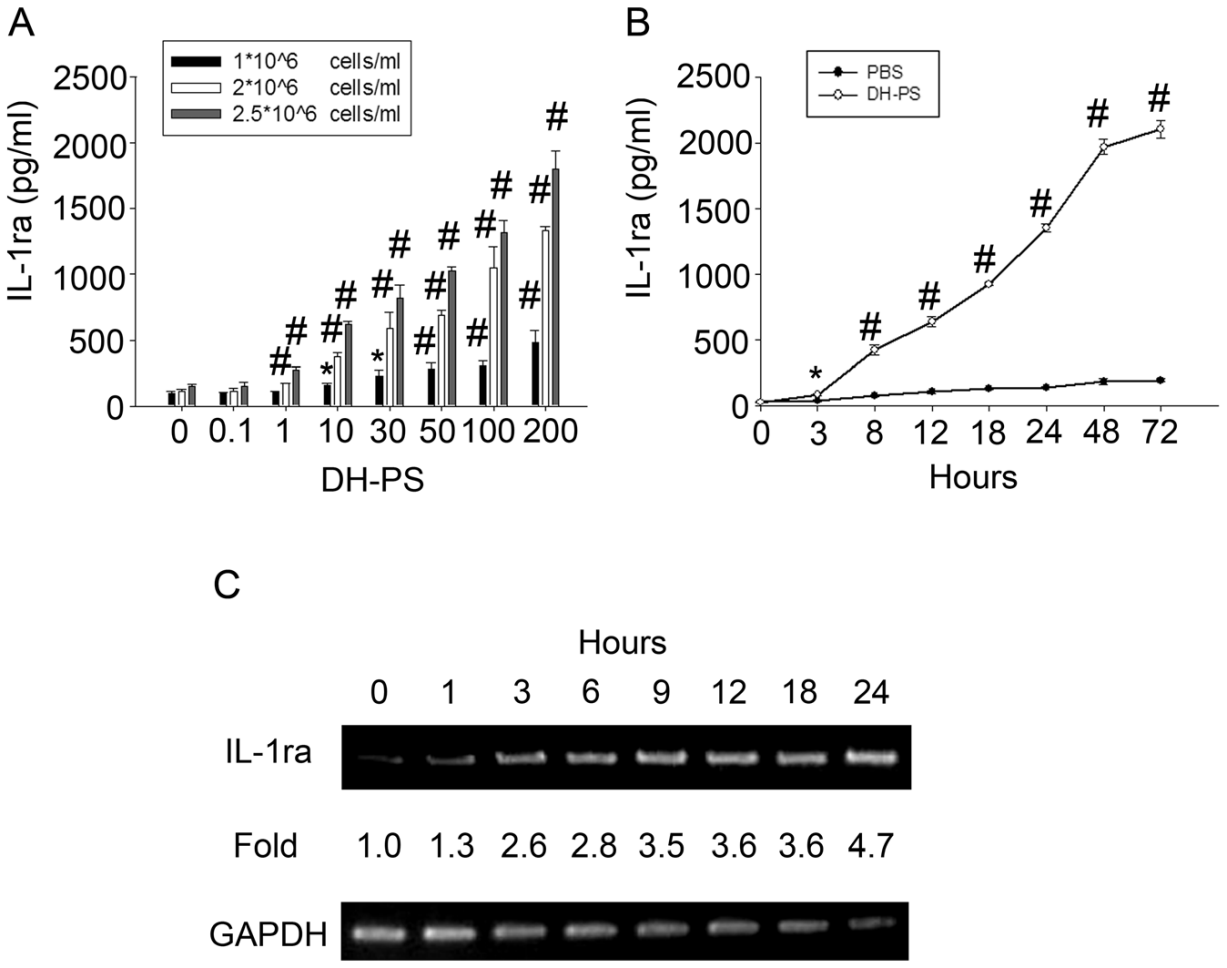
DH-PS dose-dependently induced IL-1ra production in human monocytic cell line THP-1 cells. (A) THP-1 cells were cultured with increasing concentrations of DH-PS at different cell densities for 18 hrs and supernatants were harvested for IL-1ra measurements. X-axis represented the concentration of DH-PS (μg/ml). Concentration 0 represented the use of PBS only as vehicle control. (B) THP-1 cells were cultured (2×10^6^ cells/ml) with DH-PS (100 μg/ml) or PBS and supernatants were collected in indicated time points for IL-1ra measurements. Results were presented as mean values with error bars showing the standard deviation of triplicate. Statistically significant difference: * compared with PBS-treated group, p<0.05. # compared with PBS-treated group, p<0.005. (C) THP-1 cells were cultured (2×10^6^ cells/ml) with DH-PS (100 μg/ml) and cells were collected in indicated time points for the assessment of mRNA expression of IL-1ra by RT-PCR.

Next, we used a human monocytic cell line, THP-1, as our model for more detailed studies. THP-1 cells were seeded at different densities and cultured with increasing concentrations of DH-PS for 18 hr. As shown in Figure 7A, the production of IL-1ra was dose-dependent starting from 1 μg/ml. The kinetics of IL-1ra induced by DH-PS was examined in THP-1 cells cultured at 2×10^6^ cells/ml with DH-PS (100 μg/ml) or PBS as control. The supernatants were collected for IL-1ra measurement at various time points. As shown in Figure 7B, DH-PS induced 2.2 fold increases of IL-1ra production at 3 hrs and the induction steadily increased to 72 hrs. The kinetics of IL-1ra induced by DH-PS at the RNA level was also examined by RT-PCR. As shown in Figure 7C, IL-1ra mRNA became detectable at 1 hr and appeared to reach the plateau at 12 hrs. In addition to the induction of IL-1ra in THP-1 cells, there was a dose-dependent increase in cell number upon incubation with increasing concentrations of DH-PS (up to 1.5 folds), suggesting that DH-PS might directly promote cell expansions besides the stimulation of cytokine secretions (Fig.S2).

### Intracellular signaling of DH-PS-induced IL-1ra production in monocytes

**Figure 8:**
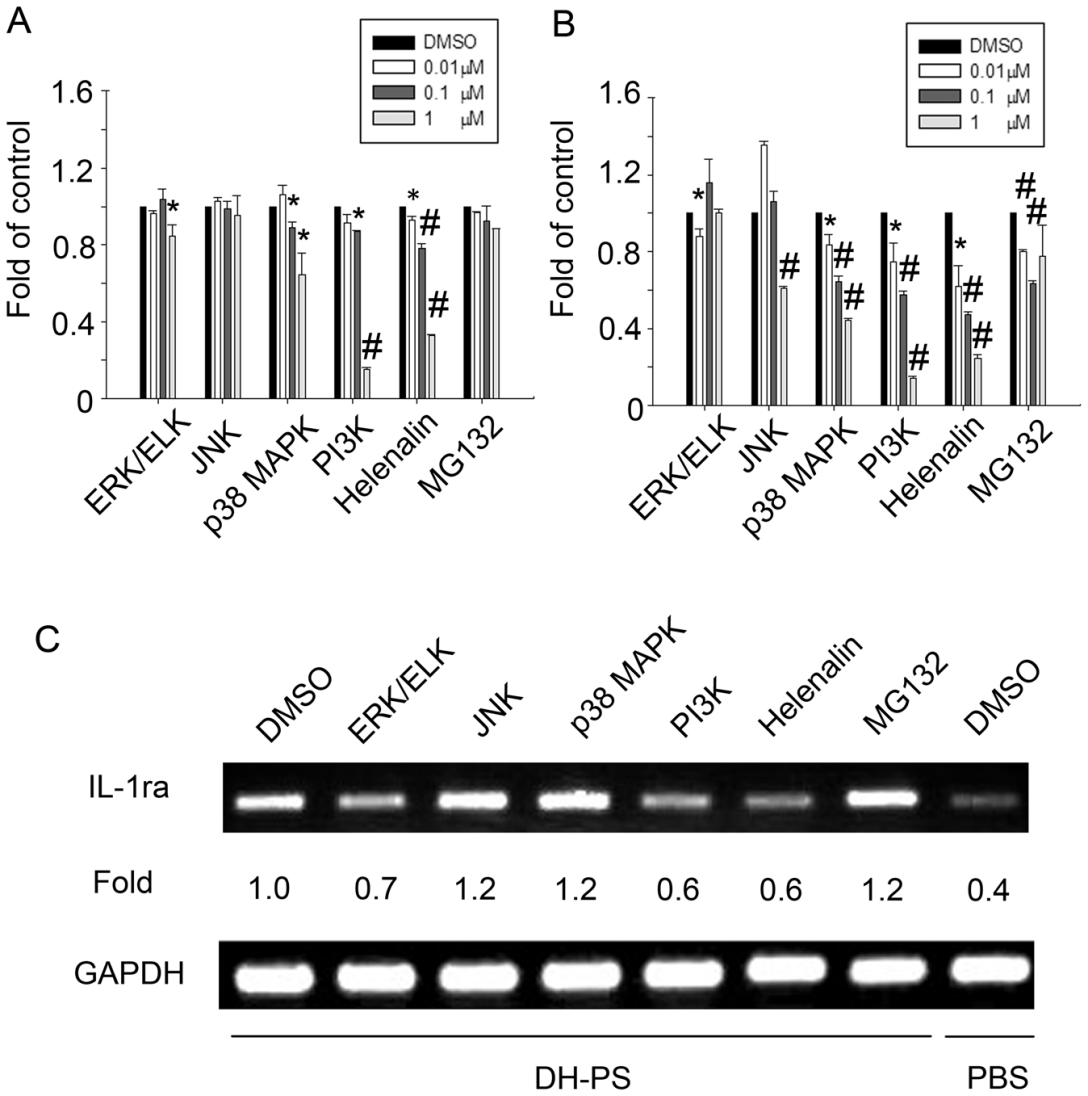
DH-PS induced IL-1ra production through MAPK, PI3K and NF-κB. (A) THP-1 cells (2×10^6^ cells/ml) were pretreated with the indicated concentrations of inhibitors for various kinases including ERK/ELK, JNK, p38 MAPK, PI3K and NFκB (0.01, 0.1 and 1 μM, except for ERK and PI3K: 0.1, 1 and 10 μM) or DMSO (0.1%) as control for 60 minutes and cultured with DH-PS (100 μg/ml) for another 18 hrs (B) Human CD14^+^ cells were pretreated with the indicated concentrations of inhibitors or DMSO as control like (A) and cultured with DH-PS (100 μg/ml) for another 18 hrs. Supernatants were harvested for IL-1ra measurements. Results were presented as fold of control (Y-axis) derived from the mean values of IL-1ra concentrations of inhibitor-treated groups divided by DMSO control group and error bars showed the standard deviation of triplicate. Statistically significant difference (Mean concentrations of IL-1ra were used for the comparisons): * compared with DMSO-treated group, p<0.05. # compared with DMSO-treated group, p<0.005. (C) THP-1 cells (2×10^6^ cells/ml) were pretreated with inhibitors for ERK/ELK (10 μM), JNK (1 μM), p38 MAPK (1 μM), PI3K (10 μM) and NFκB (1 μM) or DMSO (0.1%) as control and cultured with DH-PS (100 μg/ml) for another 12 hrs. Cells were collected for the assessment of mRNA expression of IL-1ra.

To investigate DH-PS-induced intracellular signaling mediating IL-1ra expression, we utilized inhibitors for various kinases including ERK/ELK (PD98059), JNK (SP600125), p38 MAPK (SB203580), PI3K (Ly294002) and NFκB inhibitors (Helenalin and MG132) at concentrations showing no toxicity to cells (Fig.S1). THP-1 cells (Fig.8A) or human CD14^+^ cells (Fig.8B) were pretreated with these inhibitors at increasing concentrations or DMSO as control for 60 minutes and cultured with DH-PS (100 μg/ml) for another 18 hrs. In both THP-1 and human CD14^+^ cells, the inhibitors of ERK/ELK, JNK, p38 MAPK, PI3K and NFκB diminished the secretion of IL-1ra in a dose-dependent manner. In order to determine whether these signaling molecules regulated the IL-1ra production at transcriptional level, THP-1 cells were pretreated with the inhibitors at indicated concentrations or DMSO as control for 60 minutes and cultured with DH-PS (100 μg/ml) for another 12 hrs. The mRNA expression level of IL-1ra was examined by RT-PCR. The results showed that inhibitors of ERK/ELK, PI3K and NFκB (Helenalin) dampened IL-1ra mRNA expression (Fig.8C), which was consistent with the results of IL-1ra production in protein level (Fig.8A). On the other hand, the inhibitors of JNK and p38 MAPK had no significant effects on mRNA expression.

### DH-PS induced larger amounts of IL-1ra than F3 (*G. lucidum*)

**Figure 9:**
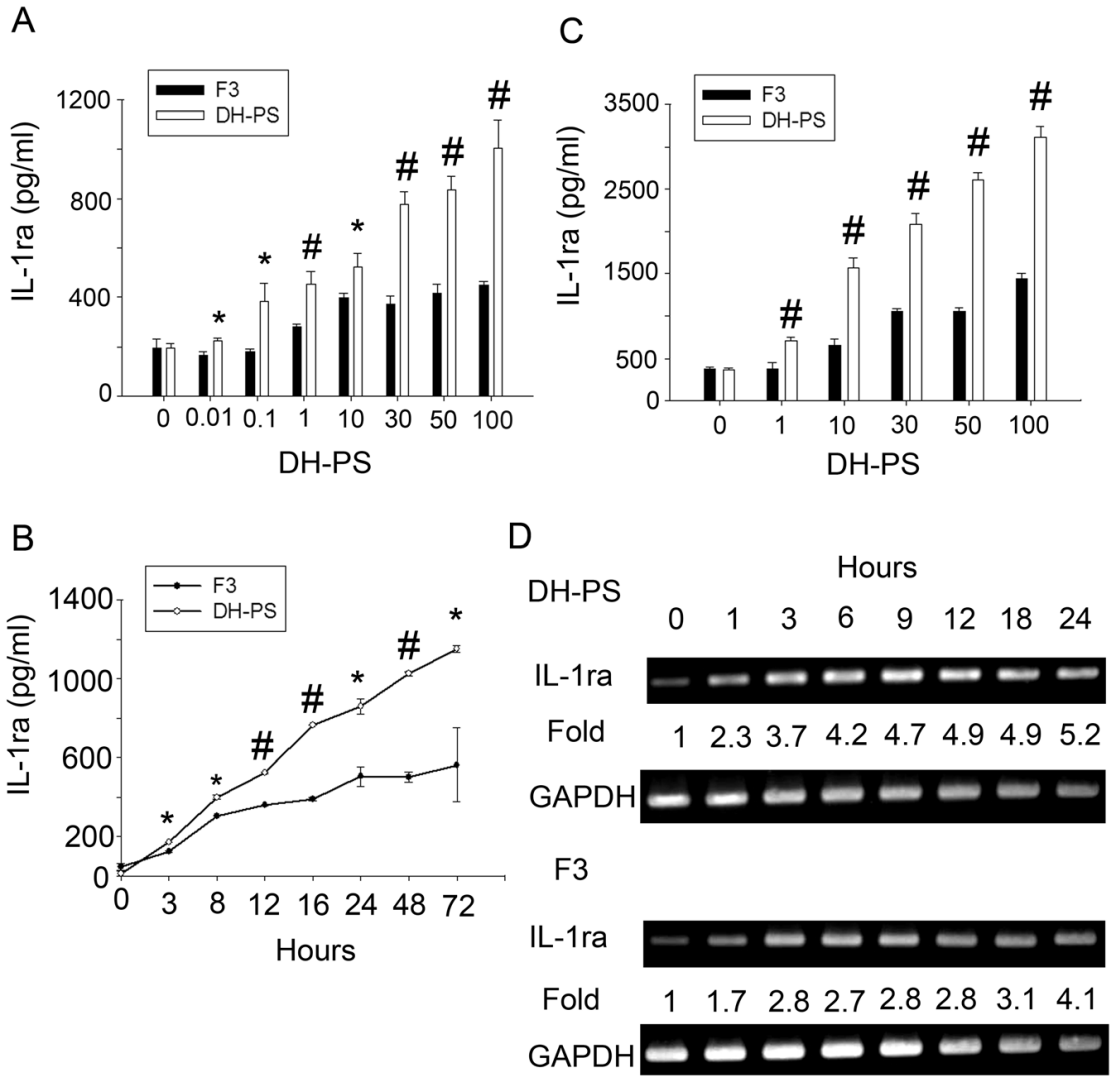
DH-PS induced more IL-1ra production than F3 in human CD14^+^ cells and THP-1 cells. (A) Human CD14^+^ cells isolated from one healthy donor were cultured (2×10^6^ cells/ml) with increasing concentrations of DH-PS or F3 for 18 hours and supernatants were collected for the measurements of IL-1ra. (B) Human CD14^+^ cells were cultured with DH-PS (100 μg/ml) or F3 (100 μg/ml) and supernatants were collected at the indicated time points for IL-1ra measurements. (C) THP-1 cells were cultured (2×10^6^ cells/ml) with increasing concentrations of DH-PS or F3 for 18 hours and supernatants were collected for the measurements of IL-1ra. In A and C, X-axis represented the concentration of DH-PS (μg/ml). Concentration 0 represented the use of PBS only as vehicle control. Results were presented as mean concentrations of IL-1ra with error bars showing the standard deviation of triplicate. Statistically significant difference: * compared with F3-treated group, p<0.05. # compared with F3-treated group, p<0.005. (D) THP-1 cells were cultured with DH-PS (100 μg/ml) or F3 (100 μg/ml) and cells were collected at the indicated time points for the assessment of mRNA expression of IL-1ra.

*Ganoderma lucidum* is a Chinese herbal medicine that has been used for centries to treat a variety of diseases including inflammation and cancer [28]. F3, the polysaccharide extract of *Ganoderma lucidum* has been reported to possess immune-modulating functions and induced IL-1ra in mice [27]. Therefore, we examined the induction of IL-1ra by DH-PS or F3 in human CD14^+^ cells and THP-1 cells and the kinetics of IL-1ra mRNA expression in THP-1 cells. Human CD14^+^ cells (Fig.9A) and THP-1 cells (Fig.9C) were cultured with increasing concentrations of DH-PS or F3 for 18 hrs. The kinetics of IL-1ra secretion in CD14^+^ cells upon the treatments of DH-PS and F3 (100 μg/ml) was also measured (Fig.9B). As shown in Figure 9A and 9C, both DH-PS and F3 induced dose-dependent productions of IL-1ra, but the maximal level induced by DH-PS was 2.2 folds of that by F3 in both CD14^+^ and THP-1 cells. As to the kinetics of IL-1ra induction in CD14^+^ cells, DH-PS elicited faster and larger amounts of IL-1ra than F3 with 1.4, 1.7 and 2.0 folds at 3, 24 and 48 hrs, respectively, reaching 2.1 folds at 72 hrs (Fig.9B). We also assessed the kinetics of IL-1ra mRNA expression in THP-1 cells cultured with DH-PS or F3. As shown in Figure 9D, DH-PS induced higher IL-1ra mRNA expression than F3, which was consistent with the ELISA data. On the other hand, F3 induced larger amounts of IL-1β (366 pg/ml, ∼52 fold increases of PBS control) (Fig.S3) than DH-PS in human CD14^+^ cells (55, 24, 70 pg/ml for 3 healthy donors respectively, Fig.4). Taken together, DH-PS-induced IL-1ra rise could over-ride IL1β-induced activity far more effectively than F3.

## Discussion

*Dendrobium huoshanense* (DH) is a versatile and valuable Chinese herbal medicine that has been used for a long period of time in China. The main bioactive molecules are polysaccharides and alkaloids. Since it is highly valuable but rare, strategies have been developed to enhance its growth and increases in polysaccharides production [29]. Using crude extracts of polysaccharides isolated from DH, we previously showed the *in vitro* induction of G-CSF and a few other cytokines in mouse splenocytes [30]. Our present study further extended the investigation to cover a comprehensive panel of cytokines and chemokines induced by DH-PS in immune cells of human *in vitro* and mouse *in vivo*. Our results clearly showed that the administration of DH-PS in mice modulated immune functions through modest activation and/or expansion of various immune cells including NK cells/activated NK cells, NKT cells/activated NKT cells, regulatory T cells, B cells/activated B cells, CD4^+^ T cells/activated CD4^+^ T cells and CD8^+^ T cells/activated CD8^+^ T cells. This was accompanied by the production of Th1 (IL-12 p40), Th2 cytokines (IL-6 and IL-10), chemokines (KC, MCP-1, MIP-1β, RANTES) and inflammatory cytokines (TNF-α). We also provided the first evidence that many cytokines and chemokines were induced by DH-PS in human PBMC (IL-1α, IL-1β, IL-12 p40, IL-6, IL-10, TNF-α, MIP-1α GM-CSF and IL-1ra) and monocytes (IL-1α, IL-1β, IL-12 p40, IL-6, IL-10, TNF-α, RANTES, MCP-1, MIP-1α, GM-CSF and IL-1ra). Notably, both IL-6 and IL-10 which have been shown to stimulate the production of IL-1ra were also induced by DH-PS. These findings were reminiscent of the potent immune-modulating effects induced by *Ganoderma lucidum* (Reishi), another popular Chinese herbal medicine. It was reported that polysaccharides isolated from *Ganoderma lucidum* (Reishi) enhanced the proliferation of Con A-stimulated mouse splenocytes [31]. We also showed that F3 stimulated the expansions of several types of immune cells including regulatory T cells [32]. But unlike DH-PS, F3 appeared to induce fewer regulatory T cells than DH-PS and larger amounts of IFN-r and IL-12 p70 which were below the detection limit upon the treatment with DH-PS, suggesting that DH-PS might exert greater anti-inflammatory activities. Taken together, DH-PS might exert its immune modulations not only by directly stimulating cytokine secretions but also through promoting the expansions and/or activations of immune cells.

Another important anti-inflammatory molecule found to be induced by DH-PS was IL-1ra which was an acute phase protein [33] and often elevated in the peripheral blood of patients with sepsis [34], chronic rheumatic diseases [35], [36], [37] and following surgical trauma [38], [39]. The beneficial effects of IL-1ra on inflammatory disorders had been demonstrated in many experimental animal models of disease by administration of recombinant IL-1ra [26], [40], [41]. In fact, recombinant IL-1ra had been in clinical use for sepsis syndrome [42], [43] and rheumatoid arthritis. Many molecules including cytokines (IL-6 and IL-10, for example) and β-glucans [26] which were the backbone components of the main bioactive polysaccharides of *G. lucidum* were reported to induce IL-1ra production. Here, we demonstrated for the first time that DH-PS induced the production of IL-1ra both *in vivo* and *in vitro*. The rapid induction of IL-1ra by DH-PS in both mouse and human suggested a direct stimulation by DH-PS. Many types of immune cells were known to secrete IL-1ra including macrophages, mast cells, neutrophils and monocytes, and our results showed that DH-PS stimulated the production of IL-1ra in monocytes but not neutrophils. We also found that unlike *in vitro* cell experiments, the level of IL-1ra in sera declined rapidly at 18 hrs after the treatment of DH-PS. This is consistent with the known short biological half-life of IL-1ra *in vivo* with rapid renal clearance and excretion in the urine [44]. In addition, DH-PS promoted THP-1 cell expansions dose-dependently, suggesting that DH-PS might also stimulate the proliferation of cells. Since certain glycans were the ligands for Toll-Like Receptors (TLRs) which were important for the activation of monocytes and involved in the transcription of IL-1ra [25], [45], it will be interesting to delineate whether and which TLRs are the receptors for DH-PS in the future.

There were several intracellular signaling mechanism reported to be involved in the regulation of IL-1ra expression. For examples, NF-κB and C/EBP had been shown to be involved in the expression of IL-1ra gene in hepatocytes [33], and MAPK (ERK1/ERK2) was associated with the IL-1ra induction stimulated by LPS [46], [47]. Serine/threonine phosphatases also participated in IL-1ra production when monocytes were contacted by stimulated T cells [48]. In addition, STAT6 was involved in IL-4 mediated IL-1ra production [49] and PI3K was reported to be essential for the IFN-β-mediated IL-1ra production [50]. Our results suggested that ERK/ELK, p38 MAPK, PI3K and NF-κB were involved in DH-PS mediated production of IL-1ra since their specific inhibitors decreased the expression of IL-1ra mRNA and the IL-1ra production in protein level. Our results suggested several possible signaling pathways involved in DH-PS-induced IL-1ra secretion.

Ganoderma lucidum (Reishi) has been known for its benefits in human health with the possession of anti-tumor and immune-modulating activities [51], [52], [53], [54]. The polysaccharides isolated from Reishi were composed of a branched (1->6)-β-D-glucan moiety. The main structure of polysaccharides isolated from DH-PS had been confirmed as acetylated glucomannan [30] which is different from Reishi. The effects of Reishi on the immune system had been attributed to cytokine inductions [55]. We compared the cytokine profiles of human monocytes cultured with DH-PS or F3 and found that both DH-PS and F3 induced several cytokines and chemokines including IL-1β, TNF-α, GM-CSF, IL-12 p40, RANTES, MCP-1, MIP-1α, IL-6 and IL-10. However, IL-12 p70 and IFN-γ were induced only by F3, suggesting a more TH1 bias activity of F3 compared with DH-PS since IL-12 p70 and IFN-γ had been reported to contribute to the differentiation of type 1 T helper cell [56]. The ability of F3 to induce the production of IL-1ra has also been reported [27]. However, we found that DH-PS induced higher IL-1ra and lower IL-1β than F3 in human CD14^+^ cells and THP-1 cells Collectively, our findings of lower levels of IL-12 p70, IFN-γ, IL-1β, larger amounts of IL-1ra and more regulatory T cells induced by DH-PS suggested that DH-PS might possess better anti-inflammatory activities than F3. Thus, DH-PS might be more potent than F3 for alleviation of inflammatory disorders. Since the main structure of DH-PS had been reported as acetylated glucomannan, it will be worthwhile to further identify the specific glycan moieties responsible for its anti-inflammatory effects and its therapeutic potential in certain immune disease models.

## Materials and Methods

### Ethics statement

Normal human blood was obtained from Taipei Blood Center with the approval of the Human Subject Research Ethics committee of both Academia Sinica and the Taiwan Blood Services Foundation. All participants provided written informed consent to Taipei Blood Services Foundation. All animal studies were performed under the approved protocol #TMIZ00JY2005158 by Institutional Animal Care and Utilization Committee of Academia Sinica.

### Preparations for crude polysaccharide extracts from *Dendrobium huoshanense* (DH-PS)

The plant material of *D. huoshanense* was obtained from Yuen-Foong-Yu Biotech Co. Taiwan [30]. Non-lignified primary mucilage polysaccharides were collected from ground leaves and stems at 4°C by dd-H_2_O extraction and the extracts were filtrated to remove the insoluble parts. The dd-H_2_O extractions (DH-PS) were dried for storage and resuspended in PBS before animal or cell experiments.

### Cell culture and reagents

Human peripheral blood mononuclear cells (PBMC) were isolated from healthy donors by Ficoll-Paque PLUS (GE Healthcare, Uppsala, Sweden) according to manufacturer's suggestions. CD14^+^ cells were further purified from PBMC by anti-CD14 microbeads and magnetically activated cell sorting (MACS) system (Miltenyi Biotech, Auburn, CA) according to manufacturer's instruction. PBMC and purified CD14^+^ cells (purity >90%) were cultured in RPMI 1640 medium (Sigma-Aldrich, USA) supplemented with 10% heat-inactivated Fetal bovine serum (FBS) (Sigma-Aldrich, USA) and penicillin/streptomycin (100 units/ml) (Invitrogen, CA, USA). For isolation of neutrophils from healthy donors, leukocytes (including neutrophils and PBMC) were separated from red blood cells (RBC) by differential sedimentation using 1.5% dextran in PBS. Neutrophils were separated from PBMC by Ficoll-Paque PLUS gradient method and further separated from the remaining RBC in the pellet by hypotonic lysis method (purity>90%) [57]. THP-1 cells (ATCC number: TIB-202) was maintained in RPMI 1640 supplemented with 10% FBS and penicillin/streptomycin (100 units/ml). For experiments of cell signaling pathway, inhibitors including PD98059, SP600125, SB203580, LY294002, Helenalin and MG132 were purchased from Calbiochem (Merck Millipore, Germany) and dissolved in dimethyl sulfoxide (DMSO) (Sigma-Aldrich, USA) for cell experiments.

### RNA isolation and RT-PCR

Total RNA was isolated by Trizol (Invitrogen, CA, USA) according to manufacturer's protocol. RNA (1 μg) was reverse-transcribed to cDNA by ThermoScript RT-PCR system (Invitrogen, CA, USA). PCR was performed using Go Taq green master mix (Promega, USA). Primers for IL-1ra: forward primer: GGCCTCCGCAGTCACCTAATCACTCT, reverse primer: TACTACTCG TCCTCCTGGAAGTAGAA. The PCR conditions were as follows: 94°C for 1 minute followed by 35 cycles of 94°C for 1 minute, 58°C for 30 seconds, 72°C for 2 minutes and a final step of 72°C for 10 minutes.

### Animal experiments and flow cytometric analysis

Mice (Six-week-old female BALB/c, from National Laboratory Animal Center, Taiwan) were injected intraperitoneally with DH-PS (100 μg or 300 μg) in PBS or PBS only as control and sacrificed at 72 hrs for the harvest of splenocytes. For flow cytometric analysis, cells were resuspended in PBS (1×10^6^ cells/ml) containing 2% FBS and 0.1% sodium azide (Sigma-Aldrich, USA) and stained with cell surface markers including NK-1.1^+^CD3^−^ (NK cells), NK-1.1^+^CD3^−^CD69^+^ (activated NK cells), NK-1.1^+^CD3^+^ (NKT cells), NK-1.1^+^CD3^+^CD69^+^ (activated NKT cells), Ly6G^+^ (granulocytes), CD11b^+^ (monocytes and macrophages), CD11c^+^ (dendritic cells), CD11C^+^CD80^+^CD86^+^ (activated dendritic cells), B220^+^CD23^+^ (B cells), B220^+^CD23^+^CD69^+^ (activated B cells), CD3^+^CD4+ (CD4^+^ T cells), CD3^+^CD4^+^CD69^+^ (activated CD4^+^ T cells), CD3^+^CD8^+^ (CD8^+^ T cells) and CD3^+^CD8^+^CD69^+^ (activated CD8^+^ T cells). For the staining of regulatory T cells (CD4^+^CD25^+^FOXP3^+^), cells were fixed with 4% paraformaldehyde (Sigma-Aldrich, USA) in PBS for 30 minutes at room temperature and permeabilized with 1% triton x-100 (Sigma-Aldrich, USA) in PBS for another 30 minutes before staining with characteristic markers for flow cytometric analysis. After washing cells with PBS, cells were analyzed on FACS Calibur (Becton Dickinson, San Jose, CA) with CellQuest software. Antibodies included PE-conjugated NK-1.1, Ly6G, CD3, CD25, CD86, FITC-conjugated CD3, CD4, CD69, CD80 and APC-conjugated CD11b, CD11c, Foxp3. All antibodies were purchased from BD bioscience, USA.

### Measurements of cytokines, chemokines and IL-1ra

To determine whether DH-PS changed the profiles of the secretions of cytokines and/or chemokines *in vivo*, mouse sera (obtained from facial vein blood sampling, Lancet) were collected at 0 (before the injection), 2 and 18 hrs after the intraperitoneal injection of DH-PS or PBS. Cytokines and chemokines were quantified by the Beadlyte mouse 21-Plex Cytokine Detection system (Millipore, Temecula, CA). For the detection of IL-1ra, sera were quantified by Mouse IL-1ra/IL-1F3 Quantikine ELISA Kit (R&D system, USA). To determine whether DH-PS changed the profiles of the secretions of cytokines and/or chemokines in human cells, cell culture supernatants were collected at 18 hrs after DH-PS or PBS treatment. Cytokines and chemokines were quantified by the Beadlyte human 22-Plex Cytokine Detection system (Millipore, Temecula, CA). For the detection of IL-1ra, supernatants were quantified by Human IL-1ra/IL-1F3 Quantikine ELISA Kit (R&D system, USA). Beadlyte human 22-Plex Cytokine Detection system included the measurements of IL-3, IL-1α, IL-1β, IL-2, IL-12 p40, IL-12 p70, GM-CSF, IP-10, MCP-1, MIP-1α, RANTES, TNF-α, IFN-γ, IL-4, IL-5, IL-6, IL-7, IL-8, IL-10, IL-13, IL-15 and Eotaxin. Beadlyte mouse 21-Plex Cytokine Detection system included the measurements of IL-3, IL-1α, IL-1β, IL-2, IL-12 p40, IL-12 p70, GM-CSF, KC, MCP-1, MIP-1β, RANTES, TNF-α, IFN-γ, IL-4, IL-5, IL-6, IL-9, IL-10, IL-13, VEGF and IL-17.

### Validation of viability or the proliferation of THP-1 cells

Cell viability and proliferation assays were determined by adding MTS reagents to cell culture medium at 1∶5 (v/v) (Promega, USA) and incubating for another 1-4 hr as the manufacturer's suggestions. The results were observed through the detection of the absorbance at 490 nm by spectrophotometer (Molecular Devices, USA).

### Statistical analysis

The Student's t test was utilized to analyze the results of cytokine and chemokine productions, cell numbers of subpopulations of splenocytes, IL-1ra measurements, assays of the inhibitor-treated cell viability and cell proliferation. For the determination of statistical significance of results in Figure 8, S1 and S2, the mean concentrations of IL1ra (pg/ml) (Fig.8) and mean values of absorbance at 490 nm (Fig.S1, S2) were used to statistical analysis before being converted to fold of control. *P* value was considered to be significant at < 0.05. Data were expressed as the mean values ± standard deviation (S.D).

## Supporting Information

Figure S1 Kinase inhibitors were not toxic to THP-1 cells in indicated concentrations. Cells were cultured (2×10^6^ cells/ml) with inhibitors for ERK/ELK (PD98059, 10 μM), JNK (SP600125, 1 μM), p38 MAPK (SB203580, 1 μM), PI3K (Ly294002, 10 μM), NFκB (Helenalin and MG132, 1 μM) or DMSO (0.1%) as control for 18 hrs. Viability was determined by MTS assay. Results were presented as fold of control (Y-axis) derived from the mean values of absorbance at 490 nm of inhibitor-treated groups divided by DMSO control group and error bars showed the standard deviation of triplicate.
(TIF)

Figure S2 DH-PS promoted the proliferation of THP-1 cells. THP-1 cells were cultured (2×10^6^ cells/ml) with increasing concentrations of DH-PS or PBS (Concentration 0) for 18 hrs and the proliferation rate was determined by MTS assay. X-axis represented the concentration of DH-PS (μg/ml). Results were presented as fold of control derived from the mean values of absorbance at 490 nm of DH-PS-treated groups divided by PBS control group and error bars showed the standard deviation of triplicate. Statistically significant difference (Mean values of absorbance were used for the comparisons): * compared with PBS-treated group, p<0.05.
(TIF)

Figure S3 F3 elicited the productions of cytokines and chemokines in human CD14^+^ cells. Human CD14^+^ cells isolated from one healthy donor were cultured with F3 (50 μg/ml) or PBS for 18 hrs and supernatants were collected for the measurements of cytokines and chemokines. Y-axis represented the mean concentrations (Conc.) of cytokines/chemokines with error bars showing the standard deviation of triplicate. Statistically significant difference: * compared with PBS-treated group, p<0.05. # compared with PBS-treated group, p<0.005.
(TIF)
