# Supplementary figures and images for: The Multifaceted Effects of Polysaccharides Isolated from Dendrobium huoshanense on Immune Functions with the Induction of Interleukin-1 Receptor Antagonist (IL-1ra) in Monocytes
Source: PLoS One. 2014 Apr 4;9(4):e94040. doi: 10.1371/journal.pone.0094040 (PMC3976396; doi:10.1371/journal.pone.0094040)

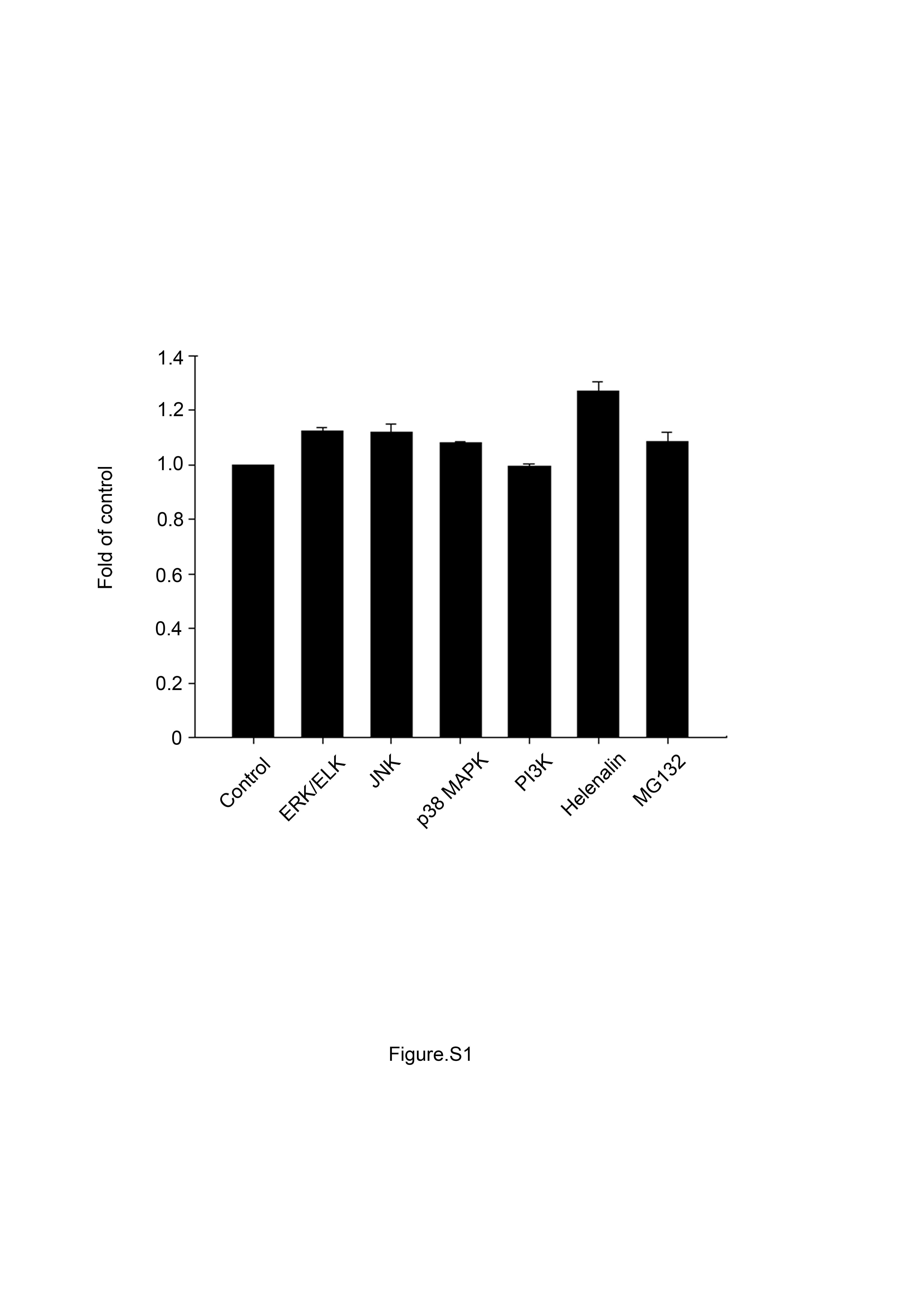

Supplement: Figure S1 — Kinase inhibitors were not toxic to THP-1 cells in indicated concentrations. Cells were cultured (2×10^6^ cells/ml) with inhibitors for ERK/ELK (PD98059, 10 μM), JNK (SP600125, 1 μM), p38 MAPK (SB203580, 1 μM), PI3K (Ly294002, 10 μM), NFκB (Helenalin and MG132, 1 μM) or DMSO (0.1%) as control for 18 hrs. Viability was determined by MTS assay. Results were presented as fold of control (Y-axis) derived from the mean values of absorbance at 490 nm of inhibitor-treated groups divided by DMSO control group and error bars showed the standard deviation of triplicate. (TIF) [file pone.0094040.s001.tif]

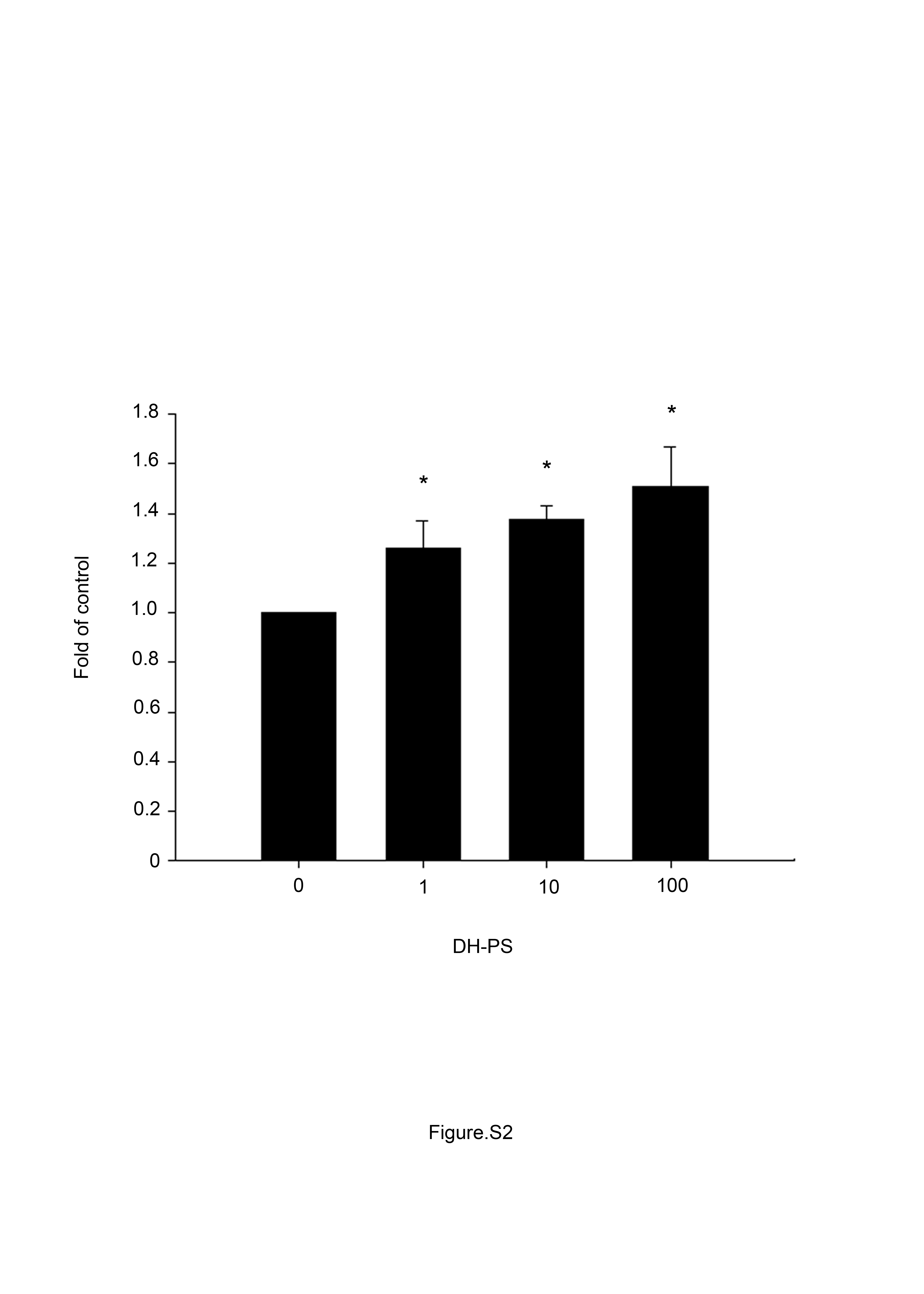

Supplement: Figure S2 — DH-PS promoted the proliferation of THP-1 cells. THP-1 cells were cultured (2×10^6^ cells/ml) with increasing concentrations of DH-PS or PBS (Concentration 0) for 18 hrs and the proliferation rate was determined by MTS assay. X-axis represented the concentration of DH-PS (μg/ml). Results were presented as fold of control derived from the mean values of absorbance at 490 nm of DH-PS-treated groups divided by PBS control group and error bars showed the standard deviation of triplicate. Statistically significant difference (Mean values of absorbance were used for the comparisons): * compared with PBS-treated group, p<0.05. (TIF) [file pone.0094040.s002.tif]

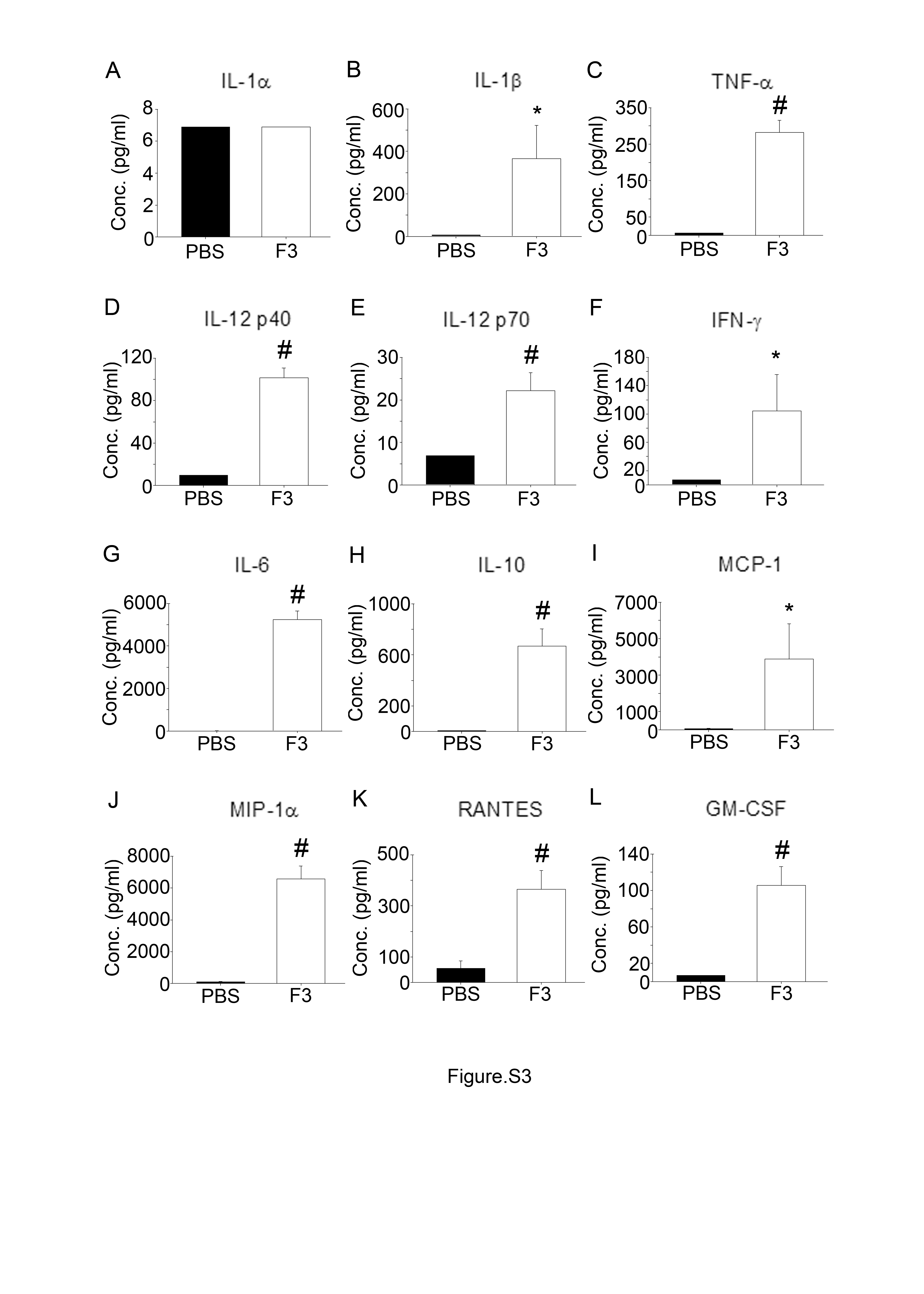

Supplement: Figure S3 — F3 elicited the productions of cytokines and chemokines in human CD14+ cells. Human CD14+ cells isolated from one healthy donor were cultured with F3 (50 μg/ml) or PBS for 18 hrs and supernatants were collected for the measurements of cytokines and chemokines. Y-axis represented the mean concentrations (Conc.) of cytokines/chemokines with error bars showing the standard deviation of triplicate. Statistically significant difference: * compared with PBS-treated group, p<0.05. # compared with PBS-treated group, p<0.005. (TIF) [file pone.0094040.s003.tif]
